# *N* ^1^,*N* ^2^-Bis­(2,6-di­methyl­phen­yl)-*N* ^1^-hydroxyformamidine *N*,*N*′-bis­(2,6-dimethyl­phen­yl)-*N*-oxidoformamidinium dichloro­methane solvate

**DOI:** 10.1107/S1600536809036708

**Published:** 2009-09-19

**Authors:** Mihaela Cibian, Sofia Derossi, Garry S. Hanan

**Affiliations:** a Département de Chimie, Université de Montréal, CP 6128, Succ. Centre-ville, Montréal, Québec, Canada H3C 3J7

## Abstract

The title compound, 2C_17_H_20_N_2_O·CH_2_Cl_2_, was obtained by *N*-oxidation of the parent formamidine with *m*-chloro-peroxy­benzoic acid (*m*-CPBA). This is the first use of the above-mentioned synthetic route for the preparation of hydroxy­amidines. The title compound crystallizes as a cyclic dimer resulting from the presence of O—H⋯O and N—H⋯N hydrogen bonds.

## Related literature

For synthesis, properties and applications of hydroxy­amidines and the parent amidines, see: Krahulic *et al.* (2005 ▶); Hirano *et al.* (2009 ▶); Coles (2006 ▶); Cotton *et al.* (2003 ▶); Chartrand & Hanan (2008 ▶); Briggs *et al.* (1976 ▶); Krajete *et al.* (2004 ▶); Kharsan & Mishra (1980 ▶); Satyanarayana & Mishra (1976 ▶). 
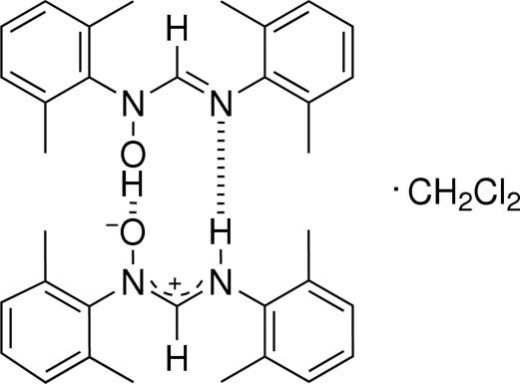


## Experimental

### 

#### Crystal data

2C_17_H_20_N_2_O·CH_2_Cl_2_
*M* *_r_* = 621.63
Orthorhombic, 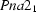

*a* = 16.360 (5) Å
*b* = 18.137 (6) Å
*c* = 11.421 (4) Å
*V* = 3388.6 (18) Å^3^
*Z* = 4
Mo *K*α radiation
μ = 0.23 mm^−1^
*T* = 200 K
0.18 × 0.09 × 0.05 mm

#### Data collection

Bruker APEXII diffractometer
Absorption correction: multi-scan (*SADABS*; Bruker, 2009 ▶) *T*_min_ = 0.806, *T*_max_ = 0.989
59541 measured reflections
6211 independent reflections
4233 reflections with *I* > 2σ(*I*)
*R*_int_ = 0.082

#### Refinement

*R*[*F* ^2^ > 2σ(*F* ^2^)] = 0.049
*wR*(*F* ^2^) = 0.136
*S* = 1.03
6211 reflections
405 parameters
1 restraint
H atoms treated by a mixture of independent and constrained refinement
Δρ_max_ = 0.24 e Å^−3^
Δρ_min_ = −0.34 e Å^−3^
Absolute structure: Flack (1983 ▶), 2931 Friedel Pairs
Flack parameter: 0.06 (9)

### 

Data collection: *APEX2* (Bruker, 2009 ▶); cell refinement: *SAINT* (Bruker, 2009 ▶); data reduction: *SAINT*; program(s) used to solve structure: *SHELXS97* (Sheldrick, 2008 ▶) and *PLATON* (Spek, 2009 ▶); program(s) used to refine structure: *SHELXL97* (Sheldrick, 2008 ▶); molecular graphics: *SHELXTL* (Sheldrick, 2008 ▶); software used to prepare material for publication: *UdMX* (Maris, 2004 ▶).

## Supplementary Material

Crystal structure: contains datablocks I, global. DOI: 10.1107/S1600536809036708/ez2181sup1.cif

Structure factors: contains datablocks I. DOI: 10.1107/S1600536809036708/ez2181Isup2.hkl

Additional supplementary materials:  crystallographic information; 3D view; checkCIF report

## Figures and Tables

**Table 1:** Hydrogen-bond geometry (Å, °)

*D*—H⋯*A*	*D*—H	H⋯*A*	*D*⋯*A*	*D*—H⋯*A*
N2—H1⋯N4^i^	0.77 (4)	2.12 (4)	2.875 (4)	166 (4)
O2—H2⋯O1^ii^	1.05 (4)	1.48 (4)	2.508 (3)	168 (3)

Symmetry codes: (i) 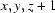; (ii) 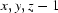.

**Fig. 1.**
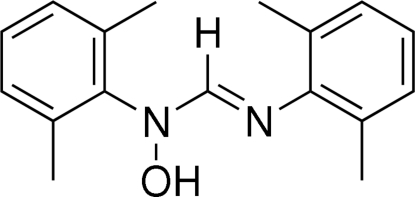
A schematic view of the N-hydroxy-N,N'-bis(2,6-dimethylphenyl)formamidine ligand.

**Fig. 2.**
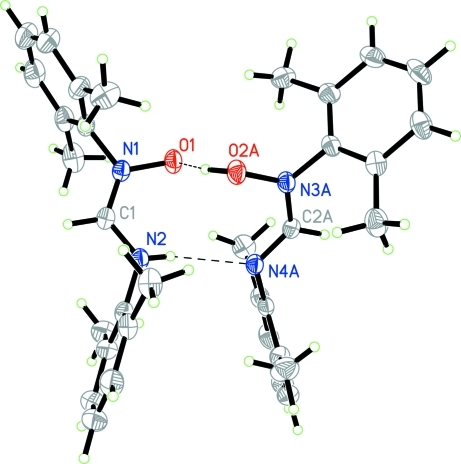
The molecular structure of the title compound, 1. Displacement ellipsoids are shown at 30% probability levels. CH2Cl2 is not shown for clarity.

## supplementary crystallographic information

### Comment

Hydroxyamidines have long been known to act as bidentate ligands that form stable 5-membered chelate rings with metal ions, and have been extensively studied as sequestrating agents for metals and in pharmacology (Kharsan & Mishra, 1980; Briggs *et al.*, 1976). However, their role as ligands for coordination and supramolecular chemistry has so far received scarce attention (Krajete *et al.*, 2004). This is somewhat surprising as they show good electronic delocalization and interesting design possibilities involving both the coordination geometry and the functionalization of the backbone. These properties make hydroxyamidines and their complexes interesting candidates for incorporation into supramolecular assemblies. In this paper we studied the behaviour of the title compound, comprising the ligand *N*-hydroxy-*N*,*N'*-bis(2,6-dimethylphenyl)formamidine in the solid state. As exemplified by Fig. 1, amidines present two sites for H-bonding interaction: the hydroxy group and the N *sp*^2^ as H-bond donor and acceptor, respectively. It is therefore plausible to expect that the molecule will dimerize in a cyclic self-complementary H-bonded O–H···N fashion.

The asymmetric unit of compound 1 presents two inequivalent molecules of 1 and one molecule of dichloromethane. The two molecules of the ligand unexpectedly form a cyclic dimer through a pair of H-bonding interactions O–H···O and one N–H···N. In the dimer, one formamidine is present in its neutral form, N=C–N–OH, while the second appears as the zwitterionic form, with the negative charge on the oxygen and the positive charge delocalized between the two nitrogen atoms, leading to two resonance forms: NH^+^=C–N–O^-^ and NH–C–N^+^=O^- ^(see scheme). Hence, the resulting bridges can be best described as O–H···O^-^ and N–H···N H-bonds. The O–H···O^-^ interaction has been previously reported in the analogous crystal structure of a benzamidine system by Krajete *et al.* (2004). In that case, however, the ligand dimerizes *via* a single H-bond. The assignment of neutral and zwitterionic moiety [N(4)–C(2)–N(3)–O(2) and N(2)–C(1)–N(1)–O(1), respectively] is consistent with the position of the hydrogen atoms observed in the crystal structure, however, none of the N–C distances has a clear character of single or double bond, ranging from 1.291 to 1.330 Å.

The planes of all the aromatic rings are tilted with respect to the O–N–C–N plane: in the neutral molecule the tilt angles are 70.5 (1) and 80.2 (1)°, while in the zwitterionic molecule these range from 87.6 (1) to 87.7 (1)°. This conformation is probably achieved to release the O–N–C–N backbone from the steric bulk of the methyl groups on the 2,6 position of the phenyl groups, and was also noticed in the structures of benzamidines studied by Krajete *et al.*(2004). The 10-atom cycle formed by the two molecules exhibits a highly distorted geometry, the planes of the N–C–N–O backbones forming an angle of 42.4 (1)°, while for the O–H···O^-^ H-bond the torsion angle N(1)–O(1)···O(2)–N(3) is 157.4 (2)°.

Structural and photophysical studies of the coordination compounds of the formamidine here described are currently in progress and will be subject of a future publication.

### Experimental

The title compound was obtained by *N*-oxidation with *m*-chloro-peroxybenzoic acid (*m*-CPBA) of the parent formamidine; the latter was prepared according to the procedure of Krahulic *et al.* (2005). To a solution of *N*,*N'*-bis(2,6-dimethylphenyl)-formamidine (1.0 g, 3.96 mmol) in 20 ml of dichloromethane, was added dropwise a solution of *m*-CPBA (0.9 g, 3.96 mmol) in 20 ml of the same solvent. The reaction mixture was stirred for 30 minutes at room temperature and successively washed with an aqueous solution of K_2_CO_3_ (5%) (2 *x* 25 ml) and of saturated NaHCO_3_ (2 *x* 25 ml). The combined organic fractions were dried over anhydrous Na_2_SO_4_ and filtered. The solvent was removed by evaporation, to afford a crude off white product. Recrystallization in DCM/ hexane (1:1) at -10°C yielded colourless X-ray quality crystals. Yield 92%.

1H NMR (DMSO-d6, 300 MHz, δ, p.p.m.): 7.80 (s, 1H), 7.20–7.10 (m, 3H), 7.00 (d, 2H), 6.88 (t, 1H), 3.50 (bs, 1H, OH), 2.31 (s,6*H*), 2.16 (s, 6H).

### Refinement

N-bound and O-bound H atoms were located in a difference Fourier map and refined. All other H atoms were placed in calculated positions, with C–H = 0.93–0.99 Å, and refined using a riding model, with *U*_iso_(H) = 1.2 or 1.5 *U*_eq_(C).

### Crystal data

**Table:**

2C_17_H_20_N_2_O·CH_2_Cl_2_	*F*(000) = 1320
*M**_r_* = 621.63	*D*_x_ = 1.218 Mg m^−^^3^
Orthorhombic, *P**n**a*2_1_	Mo *K*α radiation, λ = 0.71073 Å
Hall symbol: P 2c -2n	Cell parameters from 6035 reflections
*a* = 16.360 (5) Å	θ = 2.3–19.7°
*b* = 18.137 (6) Å	µ = 0.23 mm^−^^1^
*c* = 11.421 (4) Å	*T* = 200 K
*V* = 3388.6 (18) Å^3^	Needle, colorless
*Z* = 4	0.18 × 0.09 × 0.05 mm

### Data collection

**Table:**

Bruker APEXII diffractometer	6211 independent reflections
Radiation source: X-ray sealed tube	4233 reflections with *I* > 2σ(*I*)
Graphite	*R*_int_ = 0.082
Detector resolution: 8.3 pixels mm^-1^	θ_max_ = 25.4°, θ_min_ = 1.7°
ω scans	*h* = −19→19
Absorption correction: multi-scan (*SADABS*; Bruker, 2009)	*k* = −21→21
*T*_min_ = 0.806, *T*_max_ = 0.989	*l* = −13→13
59541 measured reflections	

### Refinement

**Table:**

Refinement on *F*^2^	Secondary atom site location: difference Fourier map
Least-squares matrix: full	Hydrogen site location: inferred from neighbouring sites
*R*[*F*^2^ > 2σ(*F*^2^)] = 0.049	H atoms treated by a mixture of independent and constrained refinement
*wR*(*F*^2^) = 0.136	*w* = 1/[σ^2^(*F*_o_^2^) + (0.067*P*)^2^ + 0.3477*P*] where *P* = (*F*_o_^2^ + 2*F*_c_^2^)/3
*S* = 1.03	(Δ/σ)_max_ < 0.001
6211 reflections	Δρ_max_ = 0.24 e Å^−^^3^
405 parameters	Δρ_min_ = −0.34 e Å^−^^3^
1 restraint	Absolute structure: Flack (1983), 2931 Friedel Pairs
Primary atom site location: structure-invariant direct methods	Flack parameter: 0.06 (9)

### Special details

**Table:**

Experimental. X-ray crystallographic data for 1 were collected from a single-crystal sample, which was mounted on a loop fiber. Data were collected using a Bruker smart diffractometer equiped with an *APEX* II CCD Detector, a graphite monochromator. The crystal-to-detector distance was 5.0 cm, and the data collection was carried out in 512 *x* 512 pixel mode. The initial unit-cell parameters were determined by a least-squares fit of the angular setting of strong reflections, collected by a 10.0 degree scan in 33 frames over four different parts of the reciprocal space (132 frames total).
Geometry. All e.s.d.'s (except the e.s.d. in the dihedral angle between two l.s. planes) are estimated using the full covariance matrix. The cell e.s.d.'s are taken into account individually in the estimation of e.s.d.'s in distances, angles and torsion angles; correlations between e.s.d.'s in cell parameters are only used when they are defined by crystal symmetry. An approximate (isotropic) treatment of cell e.s.d.'s is used for estimating e.s.d.'s involving l.s. planes.
Refinement. Refinement of *F*^2^ against ALL reflections. The weighted *R*-factor *wR* and goodness of fit *S* are based on *F*^2^, conventional *R*-factors *R* are based on *F*, with *F* set to zero for negative *F*^2^. The threshold expression of *F*^2^ > σ(*F*^2^) is used only for calculating *R*-factors(gt) *etc*. and is not relevant to the choice of reflections for refinement. *R*-factors based on *F*^2^ are statistically about twice as large as those based on *F*, and *R*- factors based on ALL data will be even larger.

### Fractional atomic coordinates and isotropic or equivalent isotropic displacement parameters (Å^2^)

**Table:**

	*x*	*y*	*z*	*U*_iso_*/*U*_eq_	
Cl1	0.79719 (8)	0.13777 (9)	0.47507 (13)	0.1095 (5)	
Cl2	0.62760 (8)	0.15036 (11)	0.53563 (14)	0.1307 (7)	
O1	0.66822 (11)	0.09653 (12)	0.9961 (2)	0.0514 (6)	
O2	0.62942 (12)	0.16297 (13)	0.1805 (2)	0.0495 (6)	
N1	0.74883 (14)	0.10368 (14)	0.9705 (2)	0.0421 (6)	
N2	0.73452 (18)	0.22848 (14)	0.9479 (2)	0.0426 (6)	
N3	0.54544 (14)	0.17397 (14)	0.1661 (2)	0.0430 (6)	
N4	0.56736 (14)	0.25572 (13)	0.0124 (2)	0.0406 (6)	
C1	0.77938 (18)	0.16788 (18)	0.9475 (3)	0.0428 (7)	
H1A	0.8360	0.1716	0.9298	0.051*	
C2	0.52018 (18)	0.22085 (17)	0.0842 (3)	0.0400 (7)	
H2A	0.4630	0.2293	0.0780	0.048*	
C11	0.79546 (18)	0.03579 (17)	0.9723 (3)	0.0460 (8)	
C12	0.8263 (2)	0.01206 (19)	1.0789 (3)	0.0545 (9)	
C13	0.8652 (2)	−0.0568 (2)	1.0819 (4)	0.0694 (11)	
H13	0.8860	−0.0753	1.1538	0.083*	
C14	0.8734 (2)	−0.0968 (2)	0.9828 (5)	0.0807 (13)	
H14	0.9015	−0.1426	0.9862	0.097*	
C15	0.8425 (2)	−0.0736 (2)	0.8775 (5)	0.0732 (12)	
H15	0.8488	−0.1038	0.8101	0.088*	
C16	0.80118 (19)	−0.0047 (2)	0.8679 (3)	0.0569 (9)	
C17	0.8183 (3)	0.0565 (2)	1.1877 (4)	0.0713 (11)	
H17A	0.8334	0.0263	1.2554	0.107*	
H17B	0.7616	0.0732	1.1962	0.107*	
H17C	0.8545	0.0995	1.1832	0.107*	
C18	0.7663 (3)	0.0240 (3)	0.7564 (4)	0.0731 (12)	
H18A	0.7066	0.0258	0.7625	0.110*	
H18B	0.7819	−0.0087	0.6918	0.110*	
H18C	0.7875	0.0737	0.7416	0.110*	
C21	0.76902 (17)	0.30074 (17)	0.9323 (3)	0.0425 (7)	
C22	0.7740 (2)	0.33000 (19)	0.8192 (3)	0.0492 (8)	
C23	0.8056 (2)	0.4009 (2)	0.8066 (4)	0.0630 (10)	
H23	0.8094	0.4220	0.7307	0.076*	
C24	0.8311 (2)	0.4405 (2)	0.9018 (4)	0.0659 (11)	
H24	0.8525	0.4887	0.8913	0.079*	
C25	0.8260 (2)	0.4108 (2)	1.0132 (4)	0.0628 (10)	
H25	0.8445	0.4387	1.0784	0.075*	
C26	0.79364 (18)	0.33988 (19)	1.0309 (3)	0.0490 (8)	
C27	0.7843 (2)	0.3081 (2)	1.1515 (3)	0.0620 (10)	
H27A	0.7271	0.2944	1.1644	0.093*	
H27B	0.8008	0.3450	1.2097	0.093*	
H27C	0.8190	0.2643	1.1591	0.093*	
C28	0.7459 (3)	0.2863 (2)	0.7147 (3)	0.0675 (11)	
H28A	0.6874	0.2757	0.7220	0.101*	
H28B	0.7764	0.2398	0.7109	0.101*	
H28C	0.7557	0.3147	0.6431	0.101*	
C31	0.49473 (18)	0.14566 (18)	0.2579 (3)	0.0412 (7)	
C32	0.48195 (19)	0.06980 (19)	0.2632 (3)	0.0474 (8)	
C33	0.4336 (2)	0.0424 (2)	0.3538 (3)	0.0583 (9)	
H33	0.4246	−0.0092	0.3602	0.070*	
C34	0.3988 (2)	0.0896 (2)	0.4340 (3)	0.0636 (11)	
H34	0.3651	0.0703	0.4944	0.076*	
C35	0.4122 (2)	0.1640 (2)	0.4277 (3)	0.0577 (9)	
H35	0.3874	0.1956	0.4837	0.069*	
C36	0.46178 (18)	0.19438 (19)	0.3404 (3)	0.0471 (8)	
C37	0.5179 (2)	0.0186 (2)	0.1726 (3)	0.0598 (9)	
H37A	0.5031	0.0359	0.0941	0.090*	
H37B	0.4965	−0.0313	0.1845	0.090*	
H37C	0.5776	0.0180	0.1804	0.090*	
C38	0.4799 (2)	0.2753 (2)	0.3355 (4)	0.0643 (10)	
H38A	0.5372	0.2827	0.3136	0.096*	
H38B	0.4698	0.2974	0.4125	0.096*	
H38C	0.4444	0.2987	0.2772	0.096*	
C41	0.52944 (17)	0.29863 (17)	−0.0770 (3)	0.0413 (7)	
C42	0.52622 (19)	0.37486 (18)	−0.0640 (3)	0.0512 (8)	
C43	0.4916 (2)	0.4163 (2)	−0.1540 (4)	0.0669 (11)	
H43	0.4888	0.4685	−0.1467	0.080*	
C44	0.4614 (2)	0.3830 (3)	−0.2536 (4)	0.0706 (12)	
H44	0.4372	0.4118	−0.3138	0.085*	
C45	0.4664 (2)	0.3084 (3)	−0.2652 (3)	0.0622 (11)	
H45	0.4461	0.2858	−0.3344	0.075*	
C46	0.50072 (19)	0.26419 (19)	−0.1777 (3)	0.0469 (8)	
C47	0.5609 (3)	0.4121 (2)	0.0424 (4)	0.0740 (11)	
H47A	0.5311	0.3956	0.1121	0.111*	
H47B	0.5554	0.4656	0.0342	0.111*	
H47C	0.6188	0.3992	0.0504	0.111*	
C48	0.5076 (3)	0.1818 (2)	−0.1925 (4)	0.0653 (10)	
H48A	0.5641	0.1664	−0.1780	0.098*	
H48B	0.4918	0.1682	−0.2725	0.098*	
H48C	0.4712	0.1572	−0.1366	0.098*	
C51	0.7056 (4)	0.1803 (4)	0.4413 (4)	0.117 (2)	
H51A	0.6905	0.1688	0.3593	0.140*	
H51B	0.7119	0.2344	0.4482	0.140*	
H1	0.688 (2)	0.229 (2)	0.962 (4)	0.064 (12)*	
H2	0.653 (2)	0.138 (2)	0.105 (4)	0.070 (11)*	

### Atomic displacement parameters (Å^2^)

**Table:**

	*U*^11^	*U*^22^	*U*^33^	*U*^12^	*U*^13^	*U*^23^
Cl1	0.0820 (8)	0.1623 (13)	0.0843 (8)	−0.0148 (8)	0.0124 (7)	−0.0280 (9)
Cl2	0.0660 (7)	0.233 (2)	0.0930 (9)	−0.0111 (9)	−0.0010 (7)	−0.0328 (11)
O1	0.0329 (11)	0.0577 (14)	0.0635 (15)	−0.0010 (9)	0.0056 (10)	−0.0061 (12)
O2	0.0333 (11)	0.0671 (15)	0.0482 (14)	0.0041 (10)	−0.0009 (10)	0.0057 (12)
N1	0.0339 (13)	0.0450 (16)	0.0474 (16)	0.0014 (12)	0.0002 (11)	−0.0052 (13)
N2	0.0332 (14)	0.0484 (17)	0.0462 (16)	0.0004 (13)	0.0021 (12)	0.0020 (12)
N3	0.0324 (13)	0.0541 (16)	0.0425 (15)	0.0037 (11)	0.0029 (11)	0.0073 (13)
N4	0.0325 (12)	0.0496 (15)	0.0399 (15)	0.0042 (11)	0.0018 (11)	0.0035 (13)
C1	0.0360 (15)	0.0491 (19)	0.0434 (19)	0.0038 (15)	−0.0012 (14)	−0.0071 (15)
C2	0.0333 (15)	0.0434 (18)	0.0432 (18)	0.0052 (14)	−0.0014 (13)	−0.0004 (15)
C11	0.0365 (15)	0.0438 (19)	0.058 (2)	0.0020 (13)	0.0077 (15)	−0.0050 (17)
C12	0.0479 (19)	0.051 (2)	0.065 (2)	0.0089 (16)	−0.0008 (17)	−0.0021 (18)
C13	0.059 (2)	0.061 (3)	0.089 (3)	0.0135 (19)	0.002 (2)	0.004 (2)
C14	0.064 (3)	0.065 (3)	0.113 (4)	0.015 (2)	0.015 (3)	−0.004 (3)
C15	0.062 (2)	0.061 (3)	0.096 (3)	−0.006 (2)	0.021 (2)	−0.036 (3)
C16	0.0474 (18)	0.061 (2)	0.062 (2)	−0.0074 (17)	0.0105 (18)	−0.022 (2)
C17	0.085 (3)	0.069 (3)	0.060 (3)	0.008 (2)	−0.015 (2)	−0.007 (2)
C18	0.064 (2)	0.098 (3)	0.058 (3)	−0.010 (2)	0.005 (2)	−0.027 (2)
C21	0.0327 (15)	0.0456 (18)	0.049 (2)	0.0018 (14)	−0.0019 (14)	−0.0080 (16)
C22	0.0483 (18)	0.046 (2)	0.053 (2)	−0.0005 (16)	0.0012 (16)	0.0014 (17)
C23	0.066 (2)	0.055 (2)	0.068 (3)	−0.0045 (19)	0.009 (2)	0.005 (2)
C24	0.064 (2)	0.048 (2)	0.086 (3)	−0.0095 (18)	0.007 (2)	−0.005 (2)
C25	0.050 (2)	0.061 (2)	0.078 (3)	−0.0065 (17)	−0.0006 (19)	−0.025 (2)
C26	0.0363 (16)	0.063 (2)	0.048 (2)	0.0008 (15)	0.0034 (15)	−0.0074 (18)
C27	0.051 (2)	0.084 (3)	0.051 (2)	−0.0011 (19)	−0.0030 (17)	−0.012 (2)
C28	0.084 (3)	0.070 (3)	0.049 (2)	−0.009 (2)	−0.0037 (19)	0.0017 (19)
C31	0.0339 (15)	0.057 (2)	0.0327 (16)	0.0000 (14)	−0.0027 (13)	0.0086 (15)
C32	0.0471 (19)	0.056 (2)	0.0391 (19)	0.0010 (15)	−0.0064 (15)	0.0115 (16)
C33	0.058 (2)	0.065 (2)	0.052 (2)	−0.0081 (18)	−0.0047 (19)	0.019 (2)
C34	0.052 (2)	0.094 (3)	0.045 (2)	−0.014 (2)	0.0003 (17)	0.026 (2)
C35	0.0485 (18)	0.085 (3)	0.0396 (19)	−0.0058 (19)	0.0024 (16)	−0.0015 (19)
C36	0.0417 (16)	0.059 (2)	0.0405 (19)	−0.0029 (15)	−0.0019 (15)	−0.0061 (16)
C37	0.071 (2)	0.052 (2)	0.057 (2)	0.0046 (18)	−0.0014 (19)	0.0001 (18)
C38	0.060 (2)	0.069 (3)	0.064 (2)	−0.0091 (19)	0.0111 (19)	−0.018 (2)
C41	0.0299 (14)	0.0482 (19)	0.0458 (19)	0.0032 (13)	0.0050 (13)	0.0102 (15)
C42	0.0420 (16)	0.049 (2)	0.062 (2)	0.0033 (15)	0.0008 (17)	0.0049 (18)
C43	0.052 (2)	0.056 (2)	0.093 (3)	0.0073 (18)	0.006 (2)	0.021 (2)
C44	0.052 (2)	0.088 (3)	0.071 (3)	0.007 (2)	−0.001 (2)	0.037 (3)
C45	0.049 (2)	0.093 (3)	0.044 (2)	−0.001 (2)	0.0002 (16)	0.018 (2)
C46	0.0398 (16)	0.059 (2)	0.0422 (19)	0.0007 (16)	−0.0011 (14)	0.0013 (17)
C47	0.081 (3)	0.053 (2)	0.088 (3)	−0.002 (2)	−0.006 (2)	−0.010 (2)
C48	0.069 (2)	0.069 (3)	0.057 (2)	−0.006 (2)	−0.0015 (19)	−0.009 (2)
C51	0.145 (5)	0.148 (5)	0.058 (3)	0.027 (4)	−0.028 (3)	−0.011 (3)

### Geometric parameters (Å, °)

**Table:**

Cl1—C51	1.729 (6)	C26—C27	1.500 (5)
Cl2—C51	1.756 (7)	C27—H27a	0.98
O1—N1	1.357 (3)	C27—H27b	0.98
O2—N3	1.398 (3)	C27—H27c	0.98
O2—H2	1.05 (4)	C28—H28a	0.98
N1—C1	1.294 (4)	C28—H28b	0.98
N1—C11	1.449 (4)	C28—H28c	0.98
N2—C1	1.322 (4)	C31—C32	1.393 (5)
N2—C21	1.438 (4)	C31—C36	1.400 (5)
N2—H1	0.77 (4)	C32—C33	1.394 (5)
N3—C2	1.330 (4)	C32—C37	1.510 (5)
N3—C31	1.433 (4)	C33—C34	1.377 (5)
N4—C2	1.291 (4)	C33—H33	0.95
N4—C41	1.426 (4)	C34—C35	1.369 (5)
C1—H1a	0.95	C34—H34	0.95
C2—H2a	0.95	C35—C36	1.398 (5)
C11—C12	1.387 (5)	C35—H35	0.95
C11—C16	1.403 (5)	C36—C38	1.498 (5)
C12—C13	1.402 (5)	C37—H37a	0.98
C12—C17	1.487 (5)	C37—H37b	0.98
C13—C14	1.351 (6)	C37—H37c	0.98
C13—H13	0.95	C38—H38a	0.98
C14—C15	1.370 (7)	C38—H38b	0.98
C14—H14	0.95	C38—H38c	0.98
C15—C16	1.426 (6)	C41—C46	1.391 (4)
C15—H15	0.95	C41—C42	1.391 (4)
C16—C18	1.488 (6)	C42—C43	1.394 (5)
C17—H17a	0.98	C42—C47	1.502 (5)
C17—H17b	0.98	C43—C44	1.380 (6)
C17—H17c	0.98	C43—H43	0.95
C18—H18a	0.98	C44—C45	1.361 (6)
C18—H18b	0.98	C44—H44	0.95
C18—H18c	0.98	C45—C46	1.399 (5)
C21—C26	1.391 (5)	C45—H45	0.95
C21—C22	1.399 (5)	C46—C48	1.508 (5)
C22—C23	1.393 (5)	C47—H47a	0.98
C22—C28	1.504 (5)	C47—H47b	0.98
C23—C24	1.369 (6)	C47—H47c	0.98
C23—H23	0.95	C48—H48a	0.98
C24—C25	1.383 (6)	C48—H48b	0.98
C24—H24	0.95	C48—H48c	0.98
C25—C26	1.406 (5)	C51—H51a	0.99
C25—H25	0.95	C51—H51b	0.99
			
N3—O2—H2	109 (2)	C22—C28—H28B	109.5
C1—N1—O1	120.3 (2)	H28A—C28—H28B	109.5
C1—N1—C11	124.4 (2)	C22—C28—H28C	109.5
O1—N1—C11	115.3 (2)	H28A—C28—H28C	109.5
C1—N2—C21	122.7 (3)	H28B—C28—H28C	109.5
C1—N2—H1	123 (3)	C32—C31—C36	122.5 (3)
C21—N2—H1	114 (3)	C32—C31—N3	118.2 (3)
C2—N3—O2	118.6 (2)	C36—C31—N3	119.3 (3)
C2—N3—C31	124.4 (2)	C31—C32—C33	118.0 (3)
O2—N3—C31	115.6 (2)	C31—C32—C37	121.3 (3)
C2—N4—C41	117.5 (2)	C33—C32—C37	120.7 (3)
N1—C1—N2	122.2 (3)	C34—C33—C32	120.5 (3)
N1—C1—H1A	118.9	C34—C33—H33	119.8
N2—C1—H1A	118.9	C32—C33—H33	119.8
N4—C2—N3	125.0 (3)	C35—C34—C33	120.7 (3)
N4—C2—H2A	117.5	C35—C34—H34	119.6
N3—C2—H2A	117.5	C33—C34—H34	119.6
C12—C11—C16	124.1 (3)	C34—C35—C36	121.3 (3)
C12—C11—N1	117.9 (3)	C34—C35—H35	119.4
C16—C11—N1	117.9 (3)	C36—C35—H35	119.4
C11—C12—C13	117.6 (3)	C35—C36—C31	117.0 (3)
C11—C12—C17	122.2 (3)	C35—C36—C38	121.8 (3)
C13—C12—C17	120.2 (4)	C31—C36—C38	121.1 (3)
C14—C13—C12	120.2 (4)	C32—C37—H37A	109.5
C14—C13—H13	119.9	C32—C37—H37B	109.5
C12—C13—H13	119.9	H37A—C37—H37B	109.5
C13—C14—C15	122.2 (4)	C32—C37—H37C	109.5
C13—C14—H14	118.9	H37A—C37—H37C	109.5
C15—C14—H14	118.9	H37B—C37—H37C	109.5
C14—C15—C16	120.8 (4)	C36—C38—H38A	109.5
C14—C15—H15	119.6	C36—C38—H38B	109.5
C16—C15—H15	119.6	H38A—C38—H38B	109.5
C11—C16—C15	115.1 (4)	C36—C38—H38C	109.5
C11—C16—C18	121.2 (3)	H38A—C38—H38C	109.5
C15—C16—C18	123.6 (4)	H38B—C38—H38C	109.5
C12—C17—H17A	109.5	C46—C41—C42	121.4 (3)
C12—C17—H17B	109.5	C46—C41—N4	119.6 (3)
H17A—C17—H17B	109.5	C42—C41—N4	118.9 (3)
C12—C17—H17C	109.5	C41—C42—C43	118.3 (3)
H17A—C17—H17C	109.5	C41—C42—C47	121.2 (3)
H17B—C17—H17C	109.5	C43—C42—C47	120.5 (3)
C16—C18—H18A	109.5	C44—C43—C42	121.1 (4)
C16—C18—H18B	109.5	C44—C43—H43	119.5
H18A—C18—H18B	109.5	C42—C43—H43	119.5
C16—C18—H18C	109.5	C45—C44—C43	119.6 (4)
H18A—C18—H18C	109.5	C45—C44—H44	120.2
H18B—C18—H18C	109.5	C43—C44—H44	120.2
C26—C21—C22	122.5 (3)	C44—C45—C46	121.6 (4)
C26—C21—N2	118.6 (3)	C44—C45—H45	119.2
C22—C21—N2	118.9 (3)	C46—C45—H45	119.2
C23—C22—C21	117.8 (3)	C41—C46—C45	118.0 (3)
C23—C22—C28	121.2 (3)	C41—C46—C48	120.9 (3)
C21—C22—C28	121.0 (3)	C45—C46—C48	121.2 (3)
C24—C23—C22	121.0 (4)	C42—C47—H47A	109.5
C24—C23—H23	119.5	C42—C47—H47B	109.5
C22—C23—H23	119.5	H47A—C47—H47B	109.5
C23—C24—C25	120.5 (3)	C42—C47—H47C	109.5
C23—C24—H24	119.7	H47A—C47—H47C	109.5
C25—C24—H24	119.7	H47B—C47—H47C	109.5
C24—C25—C26	120.7 (3)	C46—C48—H48A	109.5
C24—C25—H25	119.6	C46—C48—H48B	109.5
C26—C25—H25	119.6	H48A—C48—H48B	109.5
C21—C26—C25	117.3 (3)	C46—C48—H48C	109.5
C21—C26—C27	121.2 (3)	H48A—C48—H48C	109.5
C25—C26—C27	121.4 (3)	H48B—C48—H48C	109.5
C26—C27—H27A	109.5	CL1—C51—CL2	110.8 (3)
C26—C27—H27B	109.5	CL1—C51—H51A	109.5
H27A—C27—H27B	109.5	CL2—C51—H51A	109.5
C26—C27—H27C	109.5	CL1—C51—H51B	109.5
H27A—C27—H27C	109.5	CL2—C51—H51B	109.5
H27B—C27—H27C	109.5	H51A—C51—H51B	108.1
C22—C28—H28A	109.5		
			
O1—N1—C1—N2	0.0 (4)	N2—C21—C26—C27	−0.3 (4)
C11—N1—C1—N2	−179.4 (3)	C24—C25—C26—C21	1.3 (5)
C21—N2—C1—N1	174.6 (3)	C24—C25—C26—C27	−177.4 (3)
C41—N4—C2—N3	174.3 (3)	C2—N3—C31—C32	118.6 (3)
O2—N3—C2—N4	2.8 (5)	O2—N3—C31—C32	−75.2 (4)
C31—N3—C2—N4	168.5 (3)	C2—N3—C31—C36	−63.1 (4)
C1—N1—C11—C12	94.1 (4)	O2—N3—C31—C36	103.1 (3)
O1—N1—C11—C12	−85.3 (3)	C36—C31—C32—C33	0.8 (5)
C1—N1—C11—C16	−90.7 (4)	N3—C31—C32—C33	179.0 (3)
O1—N1—C11—C16	89.9 (3)	C36—C31—C32—C37	179.7 (3)
C16—C11—C12—C13	−0.2 (5)	N3—C31—C32—C37	−2.0 (4)
N1—C11—C12—C13	174.6 (3)	C31—C32—C33—C34	1.0 (5)
C16—C11—C12—C17	−179.5 (3)	C37—C32—C33—C34	−177.9 (3)
N1—C11—C12—C17	−4.7 (5)	C32—C33—C34—C35	−1.3 (5)
C11—C12—C13—C14	1.5 (6)	C33—C34—C35—C36	−0.3 (5)
C17—C12—C13—C14	−179.2 (4)	C34—C35—C36—C31	2.0 (5)
C12—C13—C14—C15	−2.0 (6)	C34—C35—C36—C38	−177.4 (3)
C13—C14—C15—C16	1.1 (6)	C32—C31—C36—C35	−2.2 (4)
C12—C11—C16—C15	−0.6 (5)	N3—C31—C36—C35	179.5 (3)
N1—C11—C16—C15	−175.4 (3)	C32—C31—C36—C38	177.1 (3)
C12—C11—C16—C18	179.7 (3)	N3—C31—C36—C38	−1.1 (5)
N1—C11—C16—C18	4.9 (5)	C2—N4—C41—C46	−80.7 (3)
C14—C15—C16—C11	0.2 (5)	C2—N4—C41—C42	102.9 (3)
C14—C15—C16—C18	179.9 (4)	C46—C41—C42—C43	1.4 (5)
C1—N2—C21—C26	−90.6 (4)	N4—C41—C42—C43	177.7 (3)
C1—N2—C21—C22	91.6 (4)	C46—C41—C42—C47	−177.1 (3)
C26—C21—C22—C23	0.6 (5)	N4—C41—C42—C47	−0.8 (4)
N2—C21—C22—C23	178.3 (3)	C41—C42—C43—C44	−0.1 (5)
C26—C21—C22—C28	−179.4 (3)	C47—C42—C43—C44	178.4 (3)
N2—C21—C22—C28	−1.7 (5)	C42—C43—C44—C45	−0.9 (6)
C21—C22—C23—C24	0.1 (5)	C43—C44—C45—C46	0.8 (5)
C28—C22—C23—C24	−179.9 (4)	C42—C41—C46—C45	−1.6 (4)
C22—C23—C24—C25	−0.1 (6)	N4—C41—C46—C45	−177.9 (3)
C23—C24—C25—C26	−0.7 (6)	C42—C41—C46—C48	177.6 (3)
C22—C21—C26—C25	−1.3 (5)	N4—C41—C46—C48	1.3 (4)
N2—C21—C26—C25	−179.0 (3)	C44—C45—C46—C41	0.5 (5)
C22—C21—C26—C27	177.4 (3)	C44—C45—C46—C48	−178.7 (3)

### Hydrogen-bond geometry (Å, °)

**Table:**

*D*—H···*A*	*D*—H	H···*A*	*D*···*A*	*D*—H···*A*
N2—H1···N4^i^	0.77 (4)	2.12 (4)	2.875 (4)	166 (4)
O2—H2···O1^ii^	1.05 (4)	1.48 (4)	2.508 (3)	168 (3)

Symmetry codes: (i) *x*, *y*, *z*+1; (ii) *x*, *y*, *z*−1.
